# Aquaporin 9 Represents a Novel Target of Chronic Liver Injury That May Antagonize Its Progression by Reducing Lipotoxicity

**DOI:** 10.1155/2021/5653700

**Published:** 2021-10-06

**Authors:** Quancheng Cheng, Huiru Ding, Jinyu Fang, Xuan Fang, Huaicun Liu, Jianwei Wang, Chunhua Chen, Weiguang Zhang

**Affiliations:** Department of Anatomy and Embryology, School of Basic Medical Sciences, Peking University Health Science Center, Beijing 100191, China

## Abstract

In recent years, chronic liver injury has become a common disease that harms human health. Its clinical manifestations are hepatic steatosis and secondary chronic steatohepatitis, which can quickly transform into liver fibrosis and cirrhosis if not treated in time. Therefore, this study is aimed at searching for new therapeutic targets of chronic liver injury and clarifying the molecular mechanisms of the new targets involved in chronic liver injury. After aquaporin 9 was identified as a target by proteomics, Aqp9^−/−^ mice were constructed using the CRISPR/Cas9 system. Biochemical and morphological tests were used to verify the effect of *Aqp9* knockout on early chronic liver injury. Proteomics, molecular biology, and morphology experiments were used to screen and verify the effects of *Aqp9* knockout on its downstream pathway. Through the above experiments, we demonstrated that aquaporin 9 could be used as an intervention target for antagonizing the development of early chronic liver injury and its gene knockout affected downstream inflammation, oxidative stress, apoptosis, and pyroptosis by alleviating hepatic lipotoxicity.

## 1. Introduction

Chronic liver injury (CLI) is a common disease that harms human health [1], which mainly includes alcoholic liver injury, nonalcoholic liver injury, chemical liver injury, drug liver injury, immune liver injury, and viral liver injury. Its pathogenic factors and pathogenesis are complex. When the human body is exposed to harmful substances such as CCl_4_ and alcohol, the liver is the most vulnerable organ [2]. The accumulation of free fatty acids (FFAs) and glycerol after pathogenic factor attack of hepatocytes may lead to excessive lipid accumulation and peroxidation [3], which can further induce chronic inflammation and lead to steatohepatitis [4]. Subsequently, the liver extracellular matrix is overdeposited, leading to the destruction of hepatic lobule and the formation of pseudolobules and nodules [5]. Thus, if intervention can be performed at the early stage, CLI development can be effectively curbed. However, up to now, no effective method for the early intervention of CLI has yet been reported. Therefore, clarifying the pathogenesis of early CLI and identifying new intervention targets are critical.

Hepatocytes in the early stages of CLI present steatosis, which can be effectively reversed or prevented from turning into hepatitis and fibrosis with certain interventions [6]. In the first part of this study, proteomics was used to determine the presence of lipid metabolism disorders in early CLI induced by CCl_4_, and then, aquaporin 9 (AQP9), which was speculated to play an important role in lipid metabolism, was a potential intervention target of CLI. As the main hydroglycerin channel in the liver, AQP9 facilitates the hepatic uptake of glycerol, making it available for de novo synthesis of glucose and triglyceride [7]. As a result, overexpression of AQP9 can induce steatosis in hepatocytes [8]. Furthermore, AQP9 is involved in the transformation of steatosis into chronic inflammation [7, 9]. Though Wang et al. used the short hairpin RNA of *Aqp9* to silence the *Aqp9* gene in the model of nonalcoholic fatty liver disease at the cellular level, the studies on a series of expression changes after *Aqp9* deletion were insufficient [10]. No studies have been found about the expression changes of *Aqp9* and its role in the mechanism of the early CLI. Therefore, *Aqp9* knockout (*Aqp9*^−/−^) mice were constructed and used to clarify the mechanism of AQP9 in the early CLI, which is particularly important for the study of the pathogenesis of CLI.

To date, various pathological mechanisms related to insulin resistance and metabolism have been shown to be involved in the development of CLI, in which lipotoxicity is the initiator [11]. Lipotoxicity refers to excessive accumulation of fat or an imbalance of harmful fat [12], which can activate liver inflammatory pathways, leading to chronic inflammation [4]. Lipid accumulation in hepatocytes induces *β*-oxidation of fatty acids, causing mitochondrial dysfunction and oxidative stress [13]. Proteomics was used to screen the molecular mechanisms of *Aqp9* knockout intervention in early CLI, and relevant experiments were performed to verify these results. This study is aimed at examining the influences of AQP9 on the disease course of early CLI and exploring the specific molecular mechanism of its involvement in CLI. This study also provides ideas and a theoretical basis for identifying new therapeutic targets for CLI.

## 2. Materials and Methods

### 2.1. Grouping Mice and Constructing the Early CLI Model

All procedures for this study were approved by the Ethics Committee for Animal Research Studies at the Peking University Health Science Center. Male C57BL/6 mice (20–24 g) were acclimated for at least 3 days before the experiment, with a 12-hour light/dark cycle and free to water and food.

#### 2.1.1. Part I

Sixty WT (wild-type) mice (20–24 g) were randomly divided into 2 groups, the control group and CCl_4_ group (model group), with 30 mice in each group, to screen intervention targets in the preliminary experiment.

#### 2.1.2. Part II

Sixty WT mice and sixty KO (knockout) mice (20–24 g) were divided into the WT-control group, WT-CCl_4_ group, KO-control group, and KO-CCl_4_ group, with 30 mice in each group.

Before the formal experiment, 1 cm^2^ of hair on the middle back of mice in each group was shaved to facilitate subsequent drug injection. Each group was treated as follows: the control group, WT-control group, and KO-control group were injected subcutaneously with olive oil on the back once every 3 days at 3 mL/kg body weight for 4 weeks. The CCl_4_ group, WT-CCl_4_ group, and KO-CCl_4_ group were subcutaneously injected with 40% CCl_4_ (dissolved in olive oil) on the back once every 3 days at 3 mL/kg body weight for 4 weeks.

### 2.2. Genotype Identification (DNA)

The tail tip of 20-day-old mice was disinfected with 75% alcohol, and the tail tissue of 1–3 mm was cut with surgical scissors. Next, 50 *μ*L of DNA lysate (Table 1) was added after the tissues were completely digested in a water bath at 55°C for 6–7 h, and 100 *μ*L of water was added, boiled for 5 min, oscillated on an oscillator for 1 min, and centrifuged at 10000 revolutions for 4 min. Next, centrifugation was performed at 10000 revolutions for 4 min. Polymerase chain reaction (PCR) of target genes was then performed according to the reaction procedure in Table S1 and reaction system in Table S2. The PCR products were electrophoresed in a 1% agarose gel and then observed and photographed under a UV lamp. The primer sequences used in the experiment are shown in Table S3.

### 2.3. Genotype Identification (RNA)

Total RNA was isolated from liver tissues using a FastPure cell/tissue total RNA isolation kit (Vazyme, China) according to the manufacturer's protocol. RNA was precipitated with isopropanol, washed with 75% ethanol, and dissolved in diethylpyrocarbonate-treated water. The integrity and purity of total RNA were identified by 1% agarose gel electrophoresis and with an ultraviolet spectrophotometer, respectively. Next, cDNA was synthesized according to the manufacturer's protocol of the HiScript III RT Supermix (Vazyme, China), and then, PCR of cDNA was performed according to the reaction procedure in Table S1 and the reaction system in Table S2. The PCR products were electrophoresed in a 1% agarose gel and then observed and photographed under a UV lamp. The primer sequences used in the experiment are shown in Table S3.

### 2.4. Western Blotting

Livers were collected and stored at −80°C. The livers were lysed with RIPA lysis buffer and centrifuged, and the supernatant was obtained. The protein concentration was measured using a BCA protein detection kit. Next, the proteins were separated by SDS-PAGE and transferred to Immun-Blot PVDF membranes. The membranes were washed, blocked, and incubated with the primary antibody and then with an appropriate horseradish peroxidase-conjugated secondary antibody (1 : 5000, goat anti-rabbit or goat anti-mouse; Bio-Rad Laboratories, USA). The signal was detected using an enhanced/super ECL kit. The images were scanned, and the relative density of the immunoreactive bands was determined using ImageJ software.

The primary antibodies used in this study are shown below: AQP9 (1 : 1000, AQP-009, Alomone, Israel), NLRP3 (1 : 500, A5652, ABclonal, USA), caspase-1 (1 : 500, sc-56036, Santa Cruz Biotechnology, USA), GSDMD (1 : 500, A5652, ABclonal, USA), and *β*-actin (1 : 1000, #3700,CST, USA).

### 2.5. Histomorphological Observation

The livers were collected and fixed in 10% neutral buffered formalin for histological examination. For the best fixation effect, liver tissue samples of approximately 10 mm × 5 mm × 5 mm were used. H&E, oil red O (ORO), and Masson staining were performed using standard procedures, and then, the samples were observed under a microscope. For immunohistochemistry and immunofluorescence, liver tissue sections were incubated overnight with the primary antibodies, followed by treatment with Histostain^TM^-plus kits (ZSGB-BIO, China). The final stage of immunohistochemical staining was to display the antigen-antibody complexes using a DAB substrate kit. This step of immunofluorescence staining was replaced by incubation with TRITC-labeled fluorescent secondary antibody (ZF-0316, ZSGB-BIO, China). The primary antibodies used in this study are shown as follows: AQP9 (1 : 500, AQP-009, Alomone, Israel), NLRP3 (1 : 200, A5652, ABclonal, USA), and caspase-1 p20 (1 : 200, bs-10743R, Bioss, China). Percentages of positive particles or areas visualized by microscopy were quantified by automated counting (ImageJ) on two or three fields of view selected randomly from each section. Two sections were selected from each group. The image capture was performed using a Leica DM5000 microscope.

### 2.6. Information of *Aqp9*^−/−^ Mice

The *Aqp9*^−/−^ mice were constructed in Beijing Viewsolid Biotech Co. Ltd. (Beijing, China). The transcript of *Aqp9-202* is selected for construction of KO animals. *Aqp9*^-/^- mice were constructed by using the clustered regularly interspaced short palindromic repeats (CRISPR)/CRISPR-associated (Cas) 9 system. Guide (g)RNAs direct Cas9 endonuclease cleavage of the *Aqp9* gene and create a double-strand break. Such breaks will be repaired and result in a frame shift from exon 2. Two suitable gRNAs have been found on exon 2 of *Aqp9-202*: *Aqp9*-L1: 5′-TGGATGAAGCCTAGATTCAGG-3′ and *Aqp9*-R1: 5′-TCTGATATGTAACCTGCGTGG-3′.

### 2.7. The TMT Label Quantitative Proteomics

The TMT label quantitative proteomics project was completed in cooperation with Shanghai Bioprofile Technology Company Ltd. (Shanghai, China). Liver tissues were suspended on ice in 200 *μ*L lysis buffer. Then, the samples were ultrasonicated. Undissolved cellular debris were removed by centrifugation. The supernatant was collected and quantified with a BCA Protein Assay Kit. Samples were then followed by the following steps: protein digestion, TMT labeling of peptides, high pH reversed-phase fractionation (HPRP), LC-MS analysis, database searching, and analysis (Text S1).

Two proteomics studies were performed in four groups, and 5010 proteins were identified in each group. Differentially significant expressed proteins were screened with the cutoff of a ratio fold change of >1.20 or <0.83 and *p* < 0.05.

### 2.8. Statistical Analysis

GraphPad Prism version 8.0 was used to analyze the data, and the results are presented in the graphs as means ± SEM. Single-factor analysis of variance was used for comparisons between multiple groups, followed by Dunnett's multiple comparison test. The data from two groups were analyzed by Student's *t*-test (unpaired *t*-test), and *p* < 0.05 was used to determine significant differences. Each experiment was repeated at least three times to obtain similar results.

### 2.9. Others

Serological indicators were detected by the Department of Laboratory Animal Science, Peking University Health Science Center. ELISA and enzyme activity tests were performed according to the relevant kit instructions (Dogese, China).

## 3. Results

### 3.1. Determining AQP9 as the Intervention Target of Early CLI

**Figure 1:**
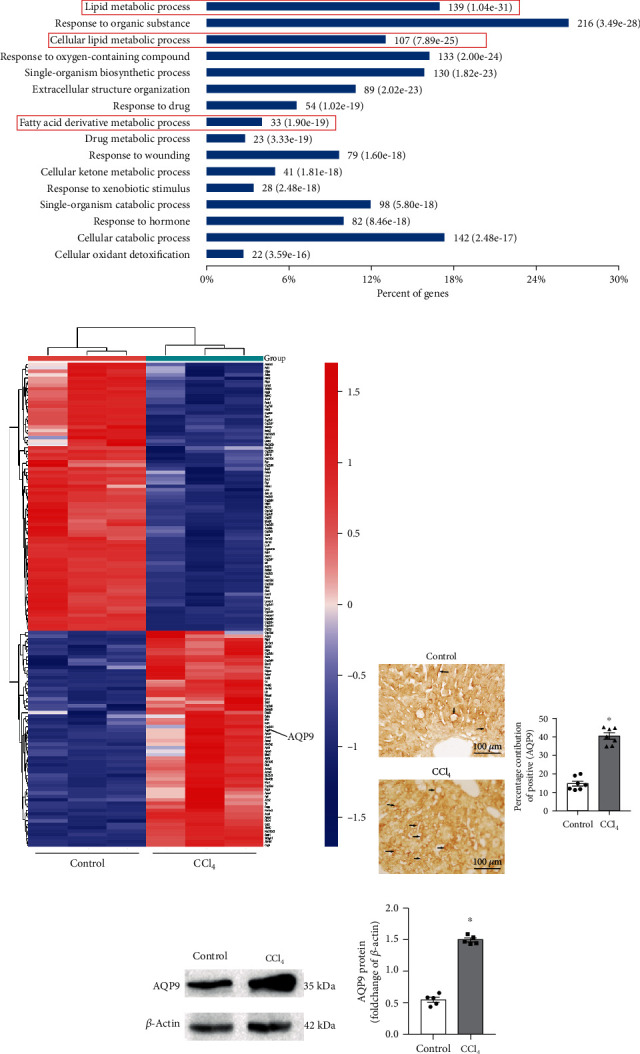
AQP9 is an intervention target for early CLI. (a) GO enrichment analysis of differential proteins in the CCl_4_ and control groups. The red boxes indicate lipid metabolism-related pathways (*n* = 3). (b) Cluster analysis of different proteins related to lipid metabolism in the CCl_4_ and control groups. Differentially significant expressed proteins were screened with the cutoff of a ratio fold change of >1.20 or <0.83 and *p* < 0.05. Red: upregulation; blue: downregulation. *n* = 3. (c) Expression changes of AQP9 after modeling (immunohistochemical staining). Arrows: AQP9-positive markers. *n* = 7. The scale bar refers to 100 *μ*m. (d) Expression changes of AQP9 after modeling (Western blotting). *n* = 5. ^∗^*p* < 0.05.

Proteomics results suggested that 617 upregulated and 266 downregulated proteins were identified in the CCl_4_ group, compared to the control group. Cluster analysis was conducted for the differential protein (Figure S1). The individual differences in the group were small, which proved that the data has certain repeatability and the differences between the groups were significant. Differential proteins were analyzed by Gene Ontology (GO) analysis. Significant changes in lipid metabolism pathways were observed in early CCl_4_-induced CLI (Figure 1(a)). To further analyze the differential proteins, we screened out the differential proteins related to lipid metabolism and constructed a heat map. Significant changes were found in the important proteins of many lipid metabolism pathways (Figure 1(b)).

The above proteomics analysis showed significant changes in lipid metabolism in early CLI induced by CCl_4_. To further study the pathogenesis of early CLI and identify new targets for intervention in early CLI, we focused our follow-up studies on glycerol channel protein AQP9, which played an important role in lipid metabolism. AQP9 is the main hydroglycerin channel protein, highly expressed in hepatocytes, which plays an important role in the synthesis of hepatic triglycerides [8]. A small number of dark-brown streaks and/or particles (AQP9-positive markers) were observed on the cell membrane in the control group, while the number was significantly increased in the CCl_4_ group (*p* < 0.05, Figure 1(c)). As shown in Figure 1(d), compared with that in the control group, the AQP9 protein in the CCl_4_ group showed a higher expression. Our statistical results also showed that the AQP9 expression in the CCl_4_ group was significantly upregulated (*p* < 0.05).

The above results confirmed that AQP9 protein expression was upregulated in early CCl_4_-induced CLI.

### 3.2. CRISPR/Cas9 Gene Editing Technology Was Used to Knock Out the *Aqp9* Gene

After determining AQP9 as the focus of the study, the CRISPR/Cas9 gene editing technology was used to knock out the *Aqp9* gene.

**Figure 2:**
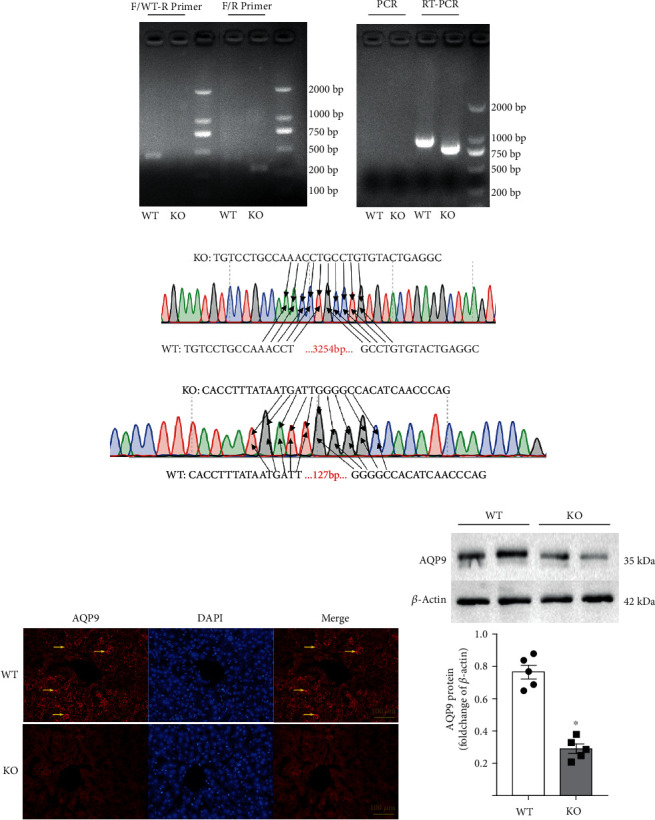
Results of genotype identification in *Aqp9*^−/−^ mice. (a) Electrophoresis results of amplified mouse tail DNA. (b) Electrophoresis results of amplified mouse liver tissue cDNA. (c) Sequencing results of PCR products of mouse tail DNA. (d) Sequencing results of RT-PCR products of mouse liver tissue mRNA. (e) Immunofluorescence staining of AQP9. Arrows: AQP9-positive markers. The scale bar refers to 100 *μ*m. (f) Western blotting results of AQP9. *n* = 5. ^∗^*p* < 0.05.

DNA from the mouse tail was extracted for PCR genotype identification. The F/R primer was used for PCR amplification, and *Aqp9*^−/−^ mice showed bands at 290 bp, while *Aqp9*^+/+^ mice showed no bands (Figure 2(a)). The F/WT-R primers were used for PCR amplification, and the *Aqp9*^−/−^ mice showed no bands, while the *Aqp9*^+/+^ mice showed bands at 430 bp (Figure 2(a)). Total RNA was extracted from liver tissue for reverse transcription-PCR (RT-PCR). The *Aqp9*^−/−^ mice showed bands at 770 bp, and the *Aqp9*^+/+^ mice showed bands at 900 bp (Figure 2(b)).

The PCR products amplified with F/R primers were sequenced and analyzed. Compared with *Aqp9*^+/+^ mice, *Aqp9*^−/−^ mice had a deletion of 3254 bp (Figure 2(c)). Sequencing and analysis of the RT-PCR products were performed. The cDNA sequence of the *Aqp9*^−/−^ mice was compared with that of the *Aqp9*^+/+^ mice, revealing that 127 bp of the *Aqp9*^−/−^ mice was missing (Figure 2(d)).

Immunofluorescence results (Figure 2(e)) showed that, compared with that of *Aqp9*^+/+^ mice, the red fluorescence signal (AQP9-positive label) in the cell membrane of the liver tissue of *Aqp9*^−/−^ mice was significantly weakened. Western blotting (Figure 2(f)) showed that AQP9 protein expression in the liver tissue of *Aqp9*^−/−^ mice was significantly decreased compared with that of *Aqp9*^+/+^ mice (*p* < 0.05).

The above results proved that *Aqp9*^−/−^ mice were successfully constructed at the gene, mRNA, and protein levels.

### 3.3. *Aqp9* Knockout Alleviates Early CLI and Lipotoxicity Induced by CCl_4_

**Figure 3:**
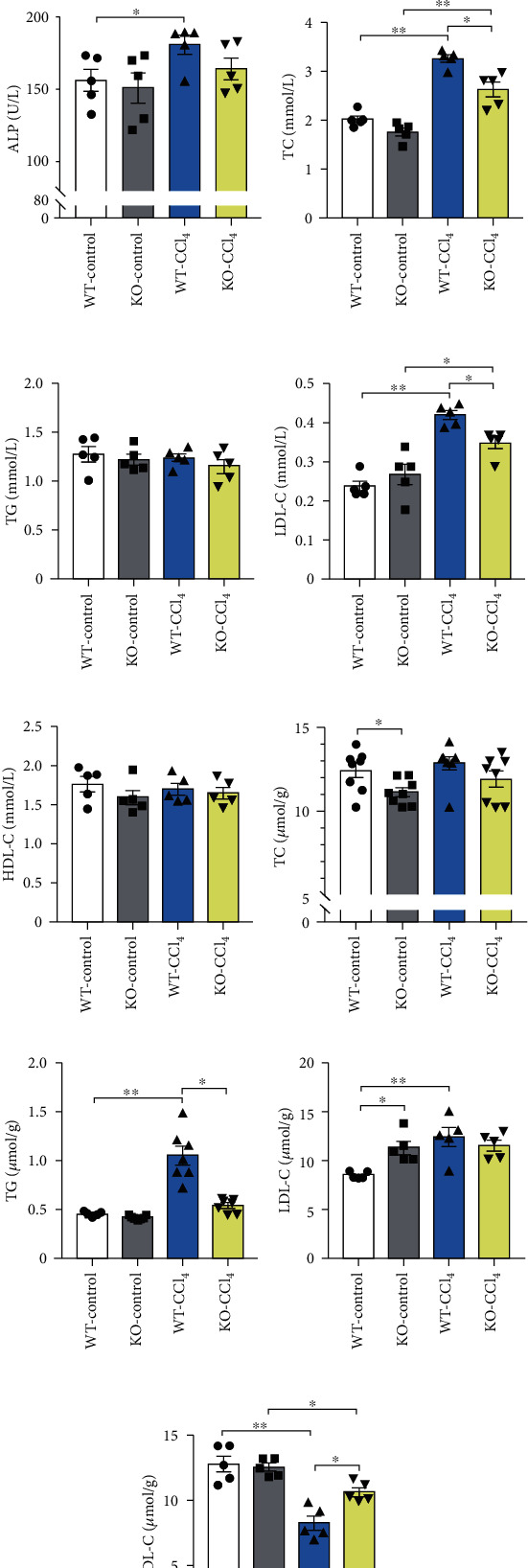
Changes in the levels of liver injury and lipid metabolism indexes. (a–c) Changes in the enzyme activity of ALT, AST, and ALP in liver tissues. (d–g) Changes in the levels of TC, TG, LDL-C, and HDL-C in serum. (h–k) Changes in the levels of TC, TG, LDL-C, and VLDL-C in liver tissues, respectively. *n* = 5. ^∗^*p* < 0.05, ^∗∗^*p* < 0.01.

The enzyme activities of alanine transaminase (ALT), aspartate aminotransferase (AST), and alkaline phosphatase (ALP) are shown in Figures 3(a)–3(c). Compared with the WT-control group, the WT-CCl_4_ group was significantly increased (ALT and AST, *p* < 0.01; ALP, *p* < 0.05). Compared with the KO-control group, the KO-CCl_4_ group was increased and ALT and AST were significantly different (*p* < 0.01). Compared with the WT-CCl_4_ group, the KO-CCl_4_ group was decreased and ALT showed statistical difference (*p* < 0.01). These results suggest that *Aqp9* knockout can improve the liver function of early CLI.

Changes in the levels of total cholesterol (TC) and low-density lipoprotein cholesterol (LDL-C) in the serum are shown in Figures 3(d) and 3(f). Compared with those in the WT-control group, their levels in the WT-CCl_4_ group were significantly higher (*p* < 0.01). Compared with the levels in the WT-CCl_4_ group, the decreased levels in the KO-CCl_4_ group were statistically significant (*p* < 0.05). The pattern of LDL-C levels in liver tissue was consistent with that in serum (Figure 3(j)). TG levels in the KO-CCl_4_ group were significantly decreased compared with those in the WT-CCl_4_ group (Figure 3(i), *p* < 0.05). Very low-density lipoprotein cholesterol (VLDL-C) in the WT-CCl_4_ group was significantly lower than that in the WT-control group (*p* < 0.01). Compared with the WT-CCl_4_ group, the KO-CCl_4_ group showed a statistically significant increase (Figure 3(k), *p* < 0.05). The above results indicate that, in early CCl_4_-induced CLI, more TG is synthesized in the liver tissue, with obstruction of VLDL-C generation in extrahepatic transport of TG. These events lead to excessive accumulation of TG in the liver tissue. However, *Aqp9* knockout can reduce the accumulation of TG and improve lipid metabolism.

**Figure 4:**
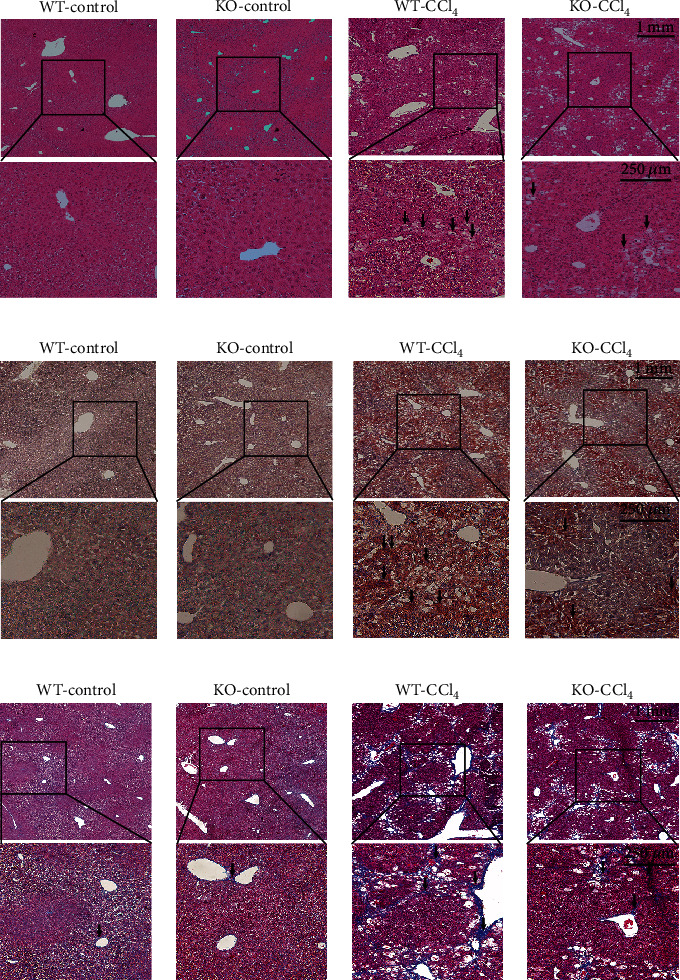
Morphological staining results. (a–c) Results of H&E, oil red O, and Masson staining. Arrows: fat deposition or collagen deposition. The long scale bar refers to 1 mm. The short scale bar refers to 250 *μ*m.

Normal liver tissue morphology was observed in the WT-control and KO-control groups. The liver tissue structure of the WT-CCl_4_ group was disordered, with no complete liver lobules or fat vacuoles throughout the field of vision. The liver tissue morphology of the KO-CCl_4_ group was damaged to a certain extent, but the liver lobule contour was still visible. Compared with the WT-CCl_4_ group, hepatic steatosis degree was significantly reduced (Figure 4(a), S2A). No red lipid droplets were observed in the WT-control and KO-control groups (Figure 4(b)). In the WT-CCl_4_ group, red lipid droplets were observed throughout the field of vision and the positive area of oil red staining was relatively large. In the KO-CCl_4_ group, red lipid droplets were significantly reduced (*p* < 0.01) (Figure 4(b), S2B). Almost no blue fiber staining was detected in the WT-control and KO-control groups. Blue fiber staining was found in the WT-CCl_4_ group. However, the blue fiber staining of the KO-CCl_4_ group was less (*p* < 0.01) (Figure 4(c), S2C).

### 3.4. Proteomics Screening of AQP9-Related Pathway Proteins

**Figure 5:**
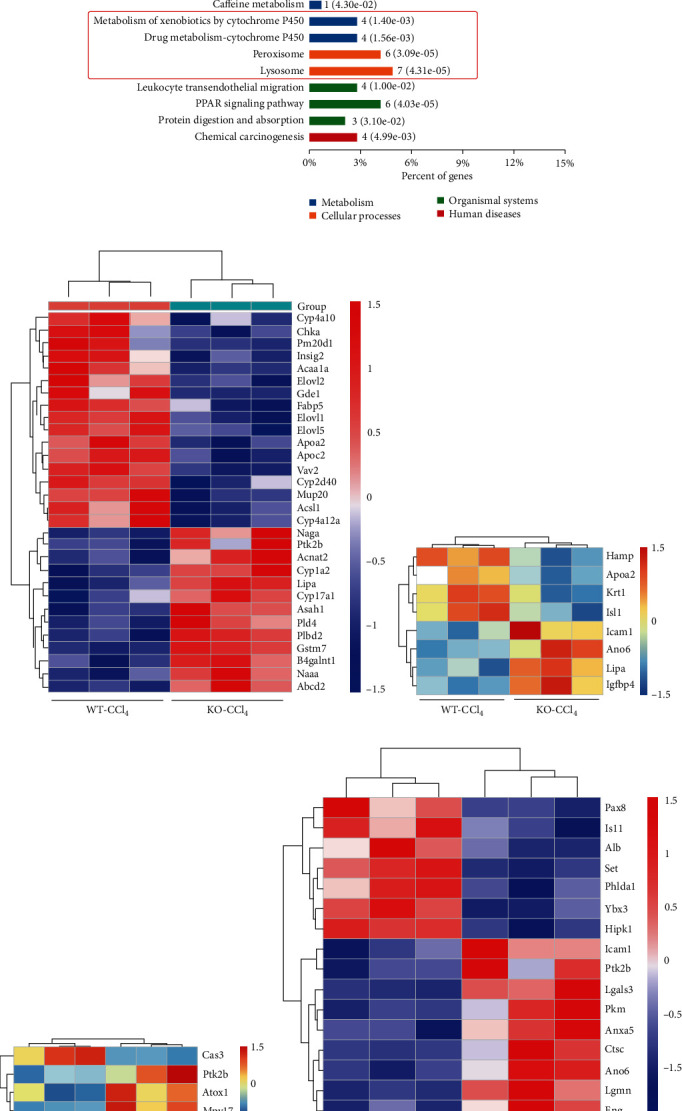
Mechanistic screening of AQP9 involved in CLI by proteomics analysis. (a) KEGG enrichment analysis of differential proteins in the KO-CCl_4_ and WT-CCl_4_ groups. (b–e) Cluster analysis of different proteins related to lipid metabolism, inflammation, oxidative stress, and apoptosis in the KO-CCl_4_ and WT-CCl_4_ groups. Differentially significant expressed proteins were screened with the cutoff of a ratio fold-change of >1.20 or <0.83 and *p* < 0.05. Red: upregulation; blue: downregulation. *n* = 3.

*Aqp9* knockout antagonized the development of early CLI to a certain extent. To further investigate the specific mechanism of *Aqp9* knockout intervention in CLI, liver tissues of the WT-CCl_4_ and KO-CCl_4_ groups were collected for proteomics experiments to screen the signaling pathways that might be involved in *Aqp9* knockout intervention in early CLI. Kyoto Encyclopedia of Genes and Genomes (KEGG) enrichment analysis was performed for the differentially expressed proteins of the KO-CCl_4_ and WT-CCl_4_ groups. *Aqp9* knockout affects lipid metabolism pathways, oxidative stress pathways, and catabolism pathways during CCl_4_-induced early CLI (Figure 5(a)). Next, different proteins related to lipid metabolism were screened out, and a small heat map was drawn (Figure 5(b)). *Aqp9* knockout affected lipid metabolism, which was consistent with AQP9-mediated glycerol entry into the liver. This finding is also consistent with that in Figure 3 showing that *Aqp9* knockout affects lipid metabolism in serum and liver tissue. Next, proteins closely related to CLI were screened, including inflammation (e.g., APOA2 and ICAM1), oxidative stress (e.g., ATOX1), and cell apoptosis (e.g., LGMN). Additionally, small heat maps were plotted (Figured 5(c)–5(e)). Because of the coexistence of inflammation and apoptosis, we hypothesized that pyroptosis was also involved in the effect of *Aqp9* knockout on early CLI.

### 3.5. Effects of *Aqp9* Knockout on the Signaling Pathways Associated with Early CLI

To further verify the results of proteomics, we conducted a series of experiments on the effects of *Aqp9* knockout on liver inflammation, oxidative stress, apoptosis, and pyroptosis.

#### 3.5.1. Influences of *Aqp9* Knockout on Inflammatory-Related Factors in Early CLI

**Figure 6:**
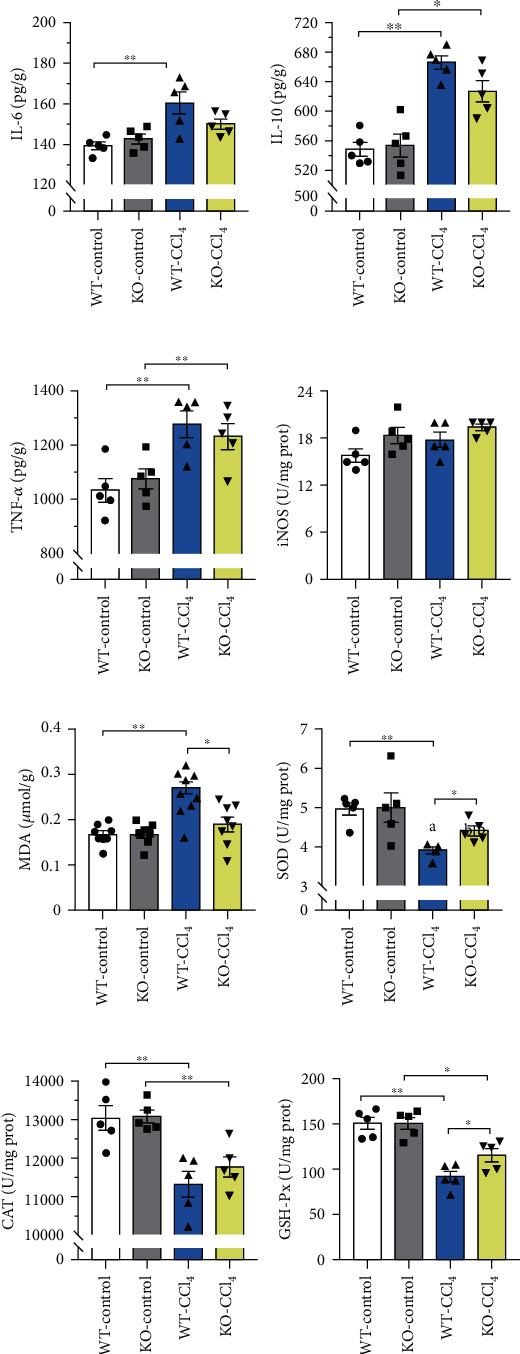
Changes in the expression of inflammatory and oxidative stress-related factors. (a–c) Changes in the expression of IL-6, IL-10, and TNF-*α* in liver tissue (ELISA). (d) Change in the enzyme activity of iNOS in liver tissues (enzyme activity detection). (e) Changes in the levels of MDA in liver tissue (ELISA). (f–h) Changes in the enzyme activity of SOD, CAT, and GSH-Px in liver tissues (enzyme activity detection). *n* = 5. ^∗^*p* < 0.05, ^∗∗^*p* < 0.01.

The expression changes of interleukin-6 (IL-6), interleukin-10 (IL-10), and tumor necrosis factor *α* (TNF-*α*) are shown in Figures 6(a)–6(c). The WT-CCl_4_ group showed significantly higher expression than the WT-control group (*p* < 0.01). Their levels in the KO-CCl_4_ group were higher than those in the KO-control group, and statistically significant differences were found in the IL-10 (*p* < 0.05) and TNF-*α* (*p* < 0.01) levels, which were decreased compared with those in the WT-CCl_4_ group, but with no significant difference. These results confirmed that *Aqp9* knockout could reduce the inflammation induced by CCl_4_.

#### 3.5.2. Effects of *Aqp9* Knockout on Oxidative Stress in Early CLI

Malondialdehyde (MDA) is an end product of membrane lipid peroxidation that reflects the degree of tissue peroxidation damage [14]. The WT-CCl_4_ group showed significantly higher MDA levels than the WT-control group (*p* < 0.01). No significant difference in the MDA levels was found in the KO-CCl_4_ group compared with that in the KO-control group, but the levels were decreased compared with that in the WT-CCl_4_ group, and the difference was statistically significant (*p* < 0.05) (Figure 6(e)). Superoxide dismutase (SOD), catalase (CAT), and glutathione peroxide (GSH-Px) are three major antioxidant enzymes in the body's oxidative stress response, reflecting the ability of tissue to resist oxidative damage [14]. The changes in enzyme activities are shown in Figures 6(f)–6(h). The activity in the WT-CCl_4_ group was significantly lower than that in the WT-control group (*p* < 0.01). The activity in the KO-CCl_4_ group was decreased compared with that in the KO-control group, and the CAT (*p* < 0.01) and GSH-Px (*p* < 0.05) levels were significantly different and showed an increased trend compared with those in WT-CCl_4_ group, among which SOD and GSH-Px showed statistically significant differences (*p* < 0.05). These results suggested that *Aqp9* knockout could reduce the CCl_4_-induced oxidative stress response.

#### 3.5.3. Effects of *Aqp9* Knockout on Apoptosis and Pyroptosis in Early CLI

**Figure 7:**
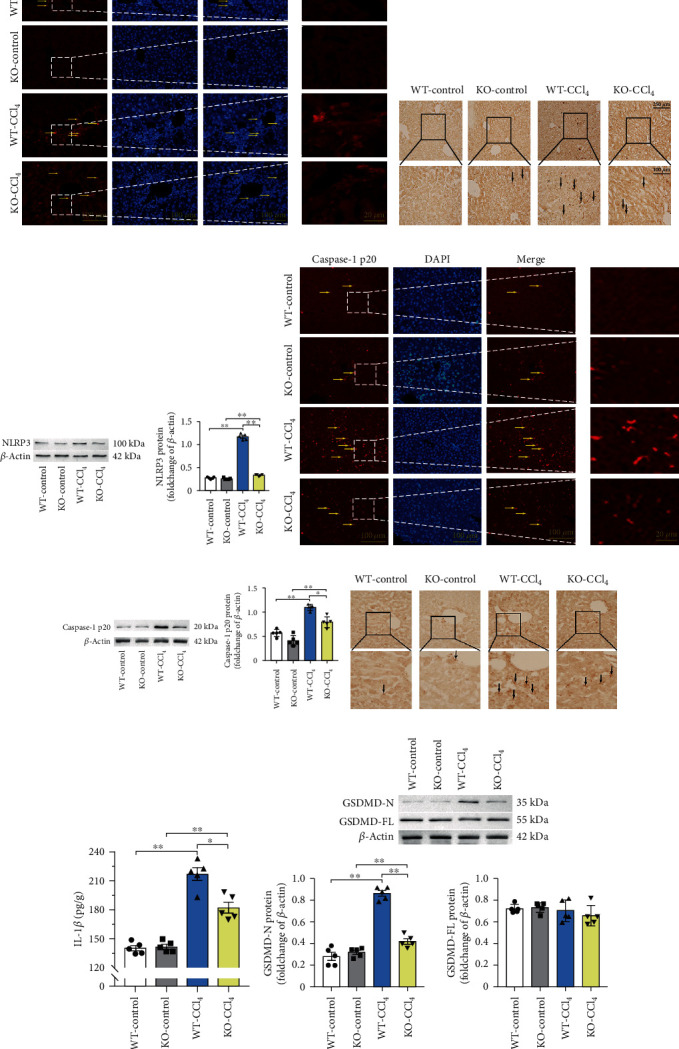
Results of apoptosis and pyroptosis. (a) TUNEL staining. Arrows: TUNEL-positive markers. (b) Immunohistochemical staining of NLRP3. Arrows: NLRP3-positive markers. (c) Western blotting of NLRP3. (d) Immunofluorescence staining of caspase-1 p20. Arrows: caspase-1 p20-positive markers. (e) Western blotting of caspase-1 p20. (f) Immunohistochemical staining of IL-1*β*. Arrows: IL-1*β*-positive markers. (g) ELISA of IL-1*β*. (h) Western blotting of GSDMD. ^∗^*p* < 0.05, ^∗∗^*p* < 0.01. *n* = 5. The long scale bar refers to 250 *μ*m. The short scale bar refers to 100 *μ*m.

TUNEL staining results are shown in Figure 7(a). No red fluorescently labeled TUNEL-positive particles were found in the WT-control and KO-control groups. More cells with TUNEL-positive granules were found in the WT-CCl_4_ group, indicating apoptosis. Although cells with TUNEL-positive granules were found in the KO-CCl_4_ group, they were significantly reduced compared with those in the WT-CCl_4_ group.

NLRP3, caspase-1, GSDMD, and interleukin-1*β* (IL-1*β*) are the four core proteins of the classical pyroptosis pathway. To verify that CCl_4_ treatment can induce pyroptosis and that *Aqp9* knockout can antagonize pyroptosis, we detected the expression of these four proteins.

No or few brown NLRP3-positive particles were detected in the cytoplasm of the WT-control and KO-control groups. The cytoplasm of the WT-CCl_4_ group showed obvious brown NLRP3-positive particles. Brown NLRP3-positive particles were decreased in the KO-CCl_4_ group (Figure 7(b)). The protein expression of NLRP3 in the WT-CCl_4_ group was significantly higher than that in the WT-control group (Figure 7(c); *p* < 0.01). The NLRP3 protein expression was decreased in the KO-CCl_4_ group than in the WT-CCl_4_ group (Figure 7(c); *p* < 0.01).

Fewer positive caspase-1 p20 particles labeled with red fluorescence were found in the WT-control and KO-control groups. More caspase-1 p20-positive particles were observed in the WT-CCl_4_ group. Caspase-1 p20-positive particles were observed in the KO-CCl_4_ group but were decreased significantly compared with those in the WT-CCl_4_ group (Figure 7(d)). The protein expression of caspase-1 p20 in the WT-CCl_4_ group was significantly higher than that in the WT-control group (Figure 7(e); *p* < 0.01). The NLRP3 protein expression was significantly decreased in the KO-CCl_4_ group compared with that in the WT-CCl_4_ group (Figure 7(e); *p* < 0.05).

Few brown IL-1*β*-positive particles were found in the WT-control and KO-control groups. In the WT-CCl_4_ group, brown IL-1*β*-positive particles were observed. Brown IL-1*β*-positive particles were decreased in the KO-CCl_4_ group (Figure 7(f)). IL-1*β* protein in the WT-CCl_4_ group was significantly increased compared with that in the WT-control group (*p* < 0.01). The IL-1*β* protein level was significantly decreased in the KO-CCl_4_ group compared with that in the WT-CCl_4_ group (Figure 7(g); *p* < 0.05).

The GSDMD-N protein (effector fragment of GSDMD) in the WT-CCl_4_ group was significantly increased compared with that in the WT-control group (*p* < 0.01). Compared with that in the WT-CCl_4_ group, GSDMD-N protein expression decreased significantly in the KO-CCl_4_ group (*p* < 0.01). No significant difference was found in the expression of GSDMD-FL (full-length GSDMD without splicing) among the four groups (Figure 7(h)).

These results suggested that *Aqp9* knockout could reduce CCl_4_-induced apoptosis and pyroptosis.

## 4. Discussion

CLI is a public health issue worldwide with an annual increase in incidence [15]. Although many methods can intervene in CLI, most are not effective in preventing the progression of chronic hepatitis to liver fibrosis and cirrhosis. Cluster analysis and GO enrichment analysis were used to determine the changes in lipid metabolism in early CLI induced by CCl_4_. This finding suggested that lipotoxicity occurred in early CLI induced by CCl_4_. To further explore the pathogenesis of early CLI, we selected the glycerol channel AQP9, which played an important role in lipid metabolism and was mainly expressed in hepatocytes, as the target for subsequent phenotypic and pathogenesis exploration.

AQP9 is the main hydroglycerin channel in hepatocytes, and its expression changes directly affect fat synthesis and gluconeogenesis. Hence, its role in nonalcoholic steatohepatitis (NASH) has been studied extensively [9, 16]. In the streptozotocin-induced diabetes model, the severity of NASH is directly proportional to the level of AQP9 in liver tissue [17]. Insulin induces phosphorylation of protein kinase B/Akt through the PI3K pathway, and Akt subsequently phosphorylates forkhead transcription factor 1 (FoxO1), isolated from insulin-response elements (IRE) which were located in the promoter region of *Aqp9* [18]. As a result, insulin negatively regulates *Aqp9* transcription [19], which explains why patients with insulin resistance are more likely to develop fatty liver disease, which can lead to CLI. Hepatic steatosis is the initial link of CLI and affects the course of subsequent diseases. Therefore, AQP9 also participates in and influences the development of early CLI. Lipotoxicity refers to excessive accumulation of fat or the production of harmful fats, leading to a series of pathological changes [12]. At the early stage of CCl_4_ modeling, lipid peroxidation and fat accumulation will occur, resulting in lipotoxicity. The mouse model of NASH induced by CCl_4_ also shows significant hepatic steatosis and inflammation [20]. These early studies suggest that CCl_4_-induced CLI initially developed and deteriorated through lipotoxicity, gradually progressing to chronic inflammation, liver fibrosis, and cirrhosis. Our study found significant lipotoxicity in serum and liver tissue lipid metabolism indexes after CCl_4_ modeling, indicating the existence of lipotoxicity in early CLI, a finding that was consistent with previous study finding. We found that the expression of AQP9 was significantly upregulated in mouse livers after 40% CCl_4_ treatment, similar to that in the results of previous basic studies in obese NASH. Under pathological conditions, the expression of AQP9 was upregulated.

In order to further explore the effect of AQP9 on CLI, the CRISPR/Cas9 gene editing technology was used to construct *Aqp9*^−/−^ mice. The gene is a genetic unit that stores information in nucleic acid molecules, and protein synthesis is completed under the guidance of genes [21]. In other words, the gene is the basis of protein expression, so the identification of DNA sequence is the most basic. If the target gene has been deleted at the DNA level confirmed by PCR and sequencing, the corresponding protein cannot be synthesized effectively due to the lack of the correct DNA translation template [22]. Therefore, the positive results of Western blotting do not necessarily mean that the protein still has function. Although *Aqp9* gene was completely knocked out, AQP9 protein, the effector, was only partially knocked out. The partial nonspecific fragments identified by Western blotting also explained the partial marginal effect of *Aqp9* knockout, which could not completely prevent the development of CLI.

The results of the liver injury index confirmed that *Aqp9* knockout could antagonize the progression of liver injury to a certain extent. We detected an increase in serum TC after modeling, but there is no significant change in liver tissue. The reason may be that excessive TC is synthesized in liver tissue and transported into blood via LDL-C so that TC in liver tissue is maintained at a relatively normal level. We reported that LDL-C in serum and liver tissue was significantly increased after modeling. This finding is consistent with the conjecture and guarantees the possibility of transshipment of large amounts of TC. Compared with the WT-CCl_4_ group, serum TC and LDL-C were decreased to a certain extent in the KO-CCl_4_ group, indicating that *Aqp9* knockout could antagonize the generation and transport of TC. After modeling, TG in serum was not significantly changed, while TG in liver tissue was significantly increased. The reason may be that TG cannot be transported out of the liver after synthesis and accumulates in the liver, causing hepatic steatosis. We subsequently detected significant downregulation of VLDL-C in extrahepatic transport of TG, further proving that CCl_4_ lead to the obstruction of TG transport and accumulation of intrahepatic TG. Compared with those of the WT-CCl_4_ group, TG and VLDL-C in liver tissue of the KO-CCl_4_ group were reversed to a certain extent, indicating that *Aqp9* knockout could antagonize the VLDL-C downregulation and TG accumulation. Morphological experiments showed that *Aqp9* knockout could reduce CCl_4_-induced liver tissue structure disorder, lipid droplets, and fibrous deposition. We confirmed that *Aqp9* knockout could antagonize the development of CLI and reduce lipotoxicity by serology, biochemical detection, and various morphological staining experiments. Rodriguez et al. reported that downregulation of AQP9 expression in obese patients played a protective role as the compensatory mechanism [9]. Wang et al. reported that aquaporin-9 downregulation prevents steatosis in oleic acid-induced nonalcoholic fatty liver disease cell models [10]. These studies all suggest that downregulation of AQP9 can reduce liver lipotoxicity, which is consistent with the finding in this study that *Aqp9* knockout alleviates CLI induced by CCl_4_.

After *Aqp9* knockout was proven to reduce the lipotoxicity of CLI, proteomics was used to confirm many changes in the expression of lipid metabolism-related proteins between the KO-CCl_4_ and WT-CCl_4_ groups. KEGG enrichment analysis confirmed changes in multiple lipid metabolism processes, suggesting that in early CCl_4_-induced CLI, *Aqp9* knockout changes the lipid metabolism pathway and regulates CCl_4_-induced lipotoxicity. This finding is consistent with the functional properties of AQP9, and the results of previous studies confirm that *Aqp9* knockout reduces lipotoxicity. Therefore, it was determined that *Aqp9* knockout could regulate changes in the downstream pathway by alleviating lipotoxicity in the liver, thus improving CLI.

Obesity induced by a high-fat diet can also lead to chronic inflammation. Excess FFAs in lipotoxic hepatocytes aggravate insulin resistance and further promote the development of inflammation [23]. Classical inflammation-related factors, such as IL-6, IL-10, TNF-*α*, and iNOS, were selected for ELISA verification. IL-6, IL-10, and TNF-*α* were significantly increased after modeling, while the level in the KO-CCl_4_ group was not increased after modeling, or the level was lower than that in the WT-CCl_4_ group. CCl_4_ modeling can induce an inflammatory response, and *Aqp9* knockout can antagonize this inflammatory response.

Oxidative stress, which is highly regulated by the body, affects most cellular functions and the dynamic balance of the body [13]. As an end product of membrane lipid peroxidation, MDA reflects the degree of tissue oxidative damage. MDA increased significantly after modeling. Compared with the WT-CCl_4_ group, MDA showed a downward trend in the KO-CCl_4_ group. SOD, CAT, and GSH-Px are three major antioxidant enzymes that reflect the antioxidant capacity of tissues. After modeling, the enzyme activities decreased, while the KO-CCl_4_ group showed an increasing trend compared with that in the WT-CCl_4_ group. CCl_4_ induced oxidative stress injury in liver tissue, and *Aqp9* knockout antagonized this oxidative stress injury.

Lipid peroxidation can cause changes in membrane fluidity and permeability. Studies have found that cardiolipin, located in the mitochondrial intima, is prone to peroxidation. Cardiolipin oxidation is necessary to promote the release of apoptotic factors in the process of mitochondria-driven apoptosis [24]. These studies have shown that lipid peroxidation of membrane phospholipids has a wide range of effects through changes in membrane structure and function, as well as through protein-lipid interactions. These interactions are particularly important to regulate endogenous apoptosis pathways. Our TUNEL staining assay also demonstrated the presence of apoptosis in early CLI induced by CCl_4_. However, *Aqp9* knockout antagonized the development of apoptosis.

Pyroptosis is a new-found programmed form of cell death that is resistant to bacterial invasion. However, excessive pyroptosis may also contribute to the development of disease. The classical inflammatory pathway of pyroptosis mainly includes key proteins such as NLRP3, caspase-1, GSDMD, and IL-1*β* [25]. In recent years, pyroptosis was found to be closely related to a various liver diseases [26–29], all of which can be categorized as CLI. The hepatic lipotoxicity of CLI activates the NLRP3 inflammasome and accelerates the chronic inflammatory response. However, reducing the formation and activation of the NLRP3 inflammasome can reduce the inflammatory response and lipotoxicity caused by pyroptosis [30]. We examined the effects of CCl_4_ and *Aqp9* knockout on the expression of NLRP3, caspase-1, GSDMD, and IL-1*β*. The expression was increased after CCl_4_ modeling. However, the KO-CCl_4_ group showed a decrease compared with the WT-CCl_4_ group, confirming that CCl_4_-induced pyroptosis exists in early CLI and that *Aqp9* knockout can antagonize pyroptosis.

## 5. Conclusions

In this study, AQP9 was identified by proteomics as an intervention target for early CLI. We also demonstrated that *Aqp9* knockout reduced hepatic lipotoxicity in CLI. Liver lipotoxicity, inflammation, oxidative stress, apoptosis, and pyroptosis were observed in CCl_4_-induced early CLI, and *Aqp9* knockout had a certain effect on the changes in these pathways in CCl_4_-induced early CLI. The reliability of omics and research results was verified by a series of experiments. *Aqp9* knockout affects downstream inflammation, oxidative stress, apoptosis, and pyroptosis by alleviating hepatic lipotoxicity and then antagonizes the development of early CLI, providing a new idea for the prevention and treatment of CLI.

## Figures and Tables

**Table 1:** DNA extraction lysate formulation (pH = 7.4).

Solute	Concentration
Tris-HCl	0.1 mol/L
EDTA	5 mmol/L
NaCl	0.2 mol/L
SDS	0.2%
PK	10%

EDTA: ethylene diamine tetraacetic acid; SDS: sodium dodecyl sulfate; PK: protease K.

## Data Availability

The raw data supporting the findings of this study are available from the corresponding author on reasonable request.

## Supplementary Materials

Supplementary 1 The supplementary figures include Figure S1 and Figure S2.

Supplementary 2 The supplementary tables include Table S1, Table S2, and Table S3.

Supplementary 3 Text S1 in the supplementary text is the TMT quantification proteomics experiment method.
