# Supplementary material for: Aquaporin 9 Represents a Novel Target of Chronic Liver Injury That May Antagonize Its Progression by Reducing Lipotoxicity
Source: Oxid Med Cell Longev. 2021 Oct 6;2021:5653700. doi: 10.1155/2021/5653700 (PMC8517626; doi:10.1155/2021/5653700)
Supplement: Supplementary 1 — The supplementary figures include Figure S1 and Figure S2. [file 5653700.f1.docx]

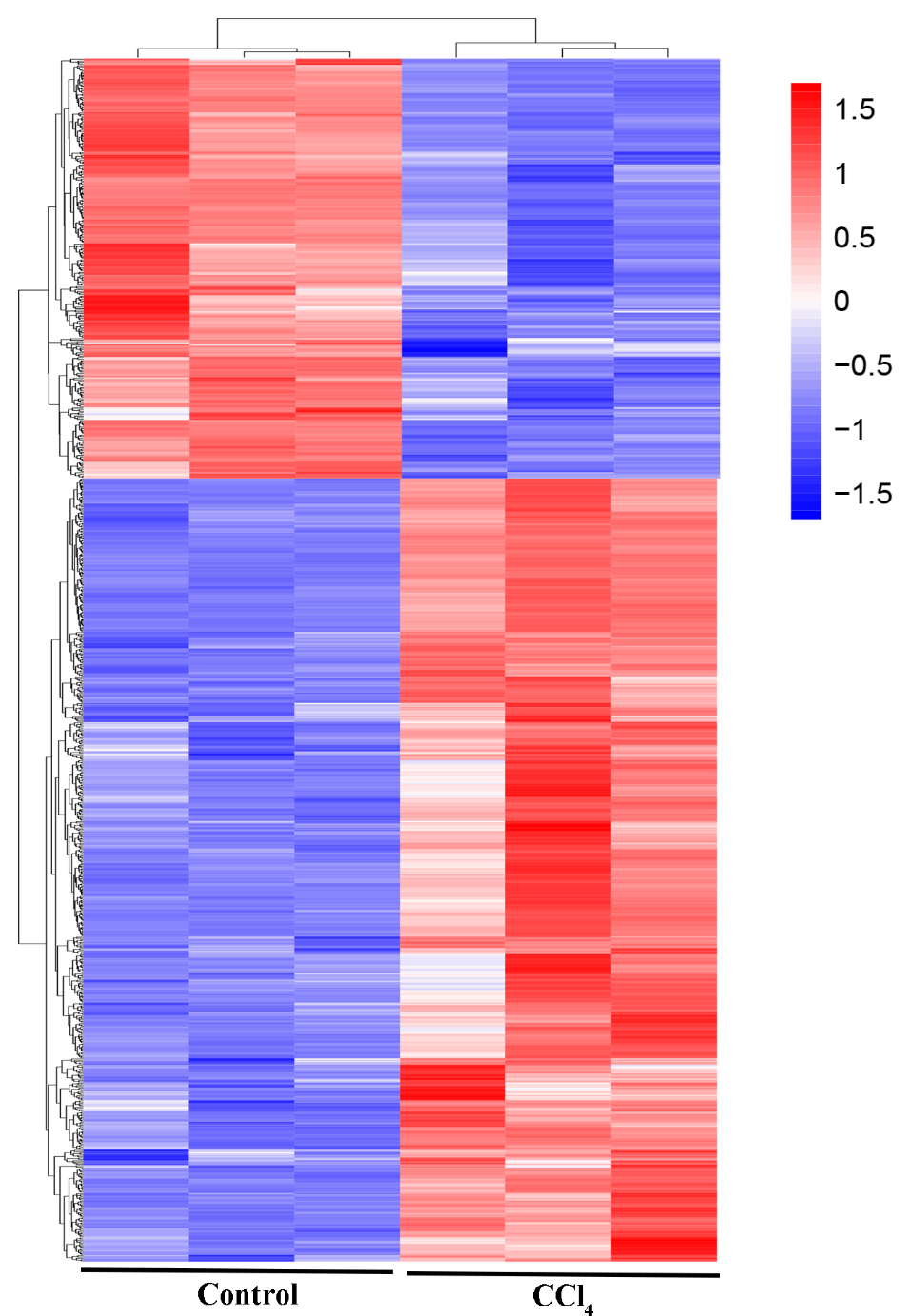


**Figure S1** Cluster analysis of all different proteins in the CCl_4_ and Control groups.


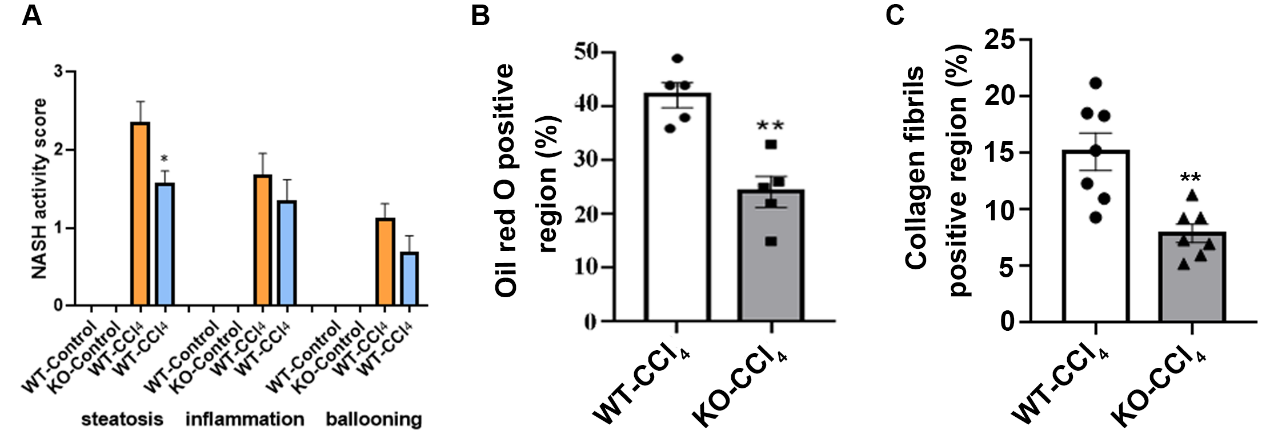


**Figure S2** Statistical results of morphological staining. (A) Nonalcoholic steatohepatitis activity scores. n=5. (B) Oil red O positive area rate. n=5. (C) Collagen fibrils positive area rate. n=7. **p*<0.05, ***p*<0.01.
