# Supplementary material for: Aquaporin 9 Represents a Novel Target of Chronic Liver Injury That May Antagonize Its Progression by Reducing Lipotoxicity
Source: Oxid Med Cell Longev. 2021 Oct 6;2021:5653700. doi: 10.1155/2021/5653700 (PMC8517626; doi:10.1155/2021/5653700)
Supplement: Supplementary 2 — The supplementary tables include Table S1, Table S2, and Table S3. [file 5653700.f2.docx]

**Table S1**: The reaction procedure of PCR

| Stage | Temperature (℃) | Time | Cycles |
| --- | --- | --- | --- |
| 1 | 94 | 2 min |  |
| 2 | 98 | 10 s | 35 |
| 3 | 60 | 30 s |  |
| 4 | 68 | 30 s |  |
| 5 | 68 | 10 min |  |
| 6 | 16 | 2 min |  |

**Table S2**: The reaction system of PCR

| Component | Volume (μl) |
| --- | --- |
| ddH2O | 9.5 |
| 2×Taq PCR Master | 12.5 |
| 5'-primer (10 μmol/L) | 1 |
| 3'-primer (10 μmol/L) | 1 |
| DNA template | 1 |

ddH_2_O, Deionized Water.

Table S3: The primer of PCR

| Purpose | Name | Sequence (5'~3') | Size (bp) |
| --- | --- | --- | --- |
| To detect *Aqp9* gene knockout | F | ATGCAGTAGCCACTTGTAGGC | KO：290 |
|  | R | ACCATATCCTGAGCTGGGCT |  |
| To amplify the WT fragment of *Aqp9* gene | F | ATGCAGTAGCCACTTGTAGGC | WT：436 |
|  | WT-R | AGCAAGGGGCATTGTAAATC |  |
| To amplify *Aqp9* cDNA | CDS-F | ATGCCTTCTGAGAAGGACCGAGCCA | WT：900 |
|  | CDS-R | CTACATGATGACGCTGAGTTCGTGT | KO：770 |
