# Supplementary material for: Aquaporin 9 Represents a Novel Target of Chronic Liver Injury That May Antagonize Its Progression by Reducing Lipotoxicity
Source: Oxid Med Cell Longev. 2021 Oct 6;2021:5653700. doi: 10.1155/2021/5653700 (PMC8517626; doi:10.1155/2021/5653700)
Supplement: Supplementary 3 — Text S1 in the supplementary text is the TMT quantification proteomics experiment method. [file 5653700.f3.docx]

**Text S1**: TMT Quantification Proteomic Experiment

**Sample preparation**

Ttissue were suspended on ice in 200 μL lysis buffer (4% SDS, 100 mM DTT, 150 mM Tris-HCl pH 8.0). Tissue were disrupted with agitation using a homogenizer, and boiling for 5min. The samples were further ultrasonicated and boiling again for another 5 min. Undissolved cellular debris were removed by centrifugation at 16000 rpm for 15 min. The supernatant were collected and quantified with a BCA Protein Assay Kit (Bio-Rad, USA).

**Protein Digestion**

Digestion of protein (200 μg for each sample) was performed according to the FASP procedure described by Wisniewski, Zougman et al. Briefly, the detergent, DTT and other low-molecular-weight components were removed using 200 μl UA buffer (8 M Urea, 150 mM Tris-HCl pH 8.0) by repeated ultrafiltration (Microcon units, 30 kD) facilitated by centrifugation. Then 100 μL 0.05 M iodoacetamide in UA buffer was added to block reduced cysteine residues and the samples were incubated for 20 min in darkness. The filter was washed with 100 μl UA buffer three times and then 100 μl 25 mM NH_4_HCO_3_ twice. Finally, the protein suspension was digested with 4 μg trypsin (Promega) in 40 μl 25 mM NH_4_HCO_3_ overnight at 37 °C, and the resulting peptides were collected as a filtrate. The peptide concentration was determined with OD280 by Nanodrop device.

**TMT Labeling of peptides**

Peptides were labeled with TMT reagents according to the manufacturer’s instructions (Thermo Fisher Scientific). Each aliquot (100 μg of peptide equivalent) was reacted with one tube of TMT reagent, respectively. After the sample was dissolved in 100 μL of 0.05M TEAB solution, pH 8.5, the TMT reagent was dissolved in 41 μL of anhydrous acetonitrile. The mixture was incubated at room temperature for 1 h. Then 8μL of 5% hydroxylamine to the sample and incubate for 15 minutes to quench the reaction. The Multiplex labeled samples were pooled together and lyophilized.

**High pH Reverse Phase Fractionation (HPRP)**

TMT-labeled peptides mixture was fractionated using a Waters XBridge BEH130 column (C18, 3.5μm, 2.1 × 150mm) on a Agilent 1290 HPLC operating at 0.3 mL/min. Buffer A consisted of 10mM ammonium formate and buffer B consisted of 10mM ammonium formate with 90% acetonitrile; both buffers were adjusted to pH 10 with ammonium hydroxide. A total of 30 fractions were collected for each peptides mixture, and then concatenated to 15 (pooling equal interval RPLC fractions). The fractions were dried for nano LC-MS/MS analysis.

**LC-MS Analysis**

LC- MS analysis were performed on a Q Exactive mass spectrometer that was coupled to Easy nLC (Thermo Fisher Scientific). Peptide from each fraction was loaded onto a the C18-reversed phase column (12cm long, 75μm ID, 3μm) in buffer A (2% acetonitrile and 0.1% Formic acid) and separated with a linear gradient of buffer B (90% acetonitrile and 0.1% Formic acid) at a flow rate of 300 nL/min over 90 min. The linear gradient was set as follows: 0–2 min, linear gradient from 2% to 5% buffer B; 2–62 min, linear gradient from 5% to 20% buffer B; 62–80 min, linear gradient from 20% to 35% buffer B; 80–83 min, linear gradient from 35% to 90% buffer B; 83–90 min, buffer B maintained at 90%. MS data was acquired using a data-dependent top15 method dynamically choosing the most abundant precursor ions from the survey scan (300–1800 m/z) for HCD fragmentation. Determination of the target value is based on predictive Automatic Gain Control (pAGC). The AGC target values of 1e6, and maximum injection time 50 ms were for full MS, and a target AGC value of 1e5, maximum injection time 100 ms for MS2. Dynamic exclusion duration was 30s. Survey scans were acquired at a resolution of 70,000 at m/z 200 and resolution for HCD spectra was set to 35,000 at m/z 200. Normalized collision energy was 30. The instrument was run with peptide recognition mode enabled.

**Database Searching and Analysis**

The resulting LC-MS/MS raw files were imported into MaxQuant software (version 1.6.0.16) for data interpretation and protein identification against the database Uniprot_Hordeum-vulgare_201747-20180125 (downloaded on 25/01/2018, and including 201747 protein sequences), which is sourced from the protein database at <https://www.uniprot.org/uniprot/?query=Hordeum-vulgare&sort=score>. An initial search was set at a precursor mass window of 6 ppm. The search followed an enzymatic cleavage rule of Trypsin/P and allowed maximal two missed cleavage sites and a mass tolerance of 20ppm for fragment ions. The modification set was as following: fixed modification: Carbamidomethyl (C), TMT6plex(K), TMT6plex(N-term), Variable modification：Oxidation(M) and Acetyl (Protein N-term). The minimum 6 amino acids for peptide, ≥1 unique peptides were required per protein. For peptide and protein identification, false discovery rate (FDR) was set to 1%. TMT reporter ion intensity were used for quantification.

**Bioinformatics analysis**

Analyses of bioinformatics data were carried out with Perseus software, Microsoft Excel and R statistical computing software. Differentially significant expressed proteins were screened with the cutoff of a ratio fold-change of >1.20 or <0.83 and P-values < 0.05. Expression data were grouped together by hierarchical clustering according to the protein level. To annotate the sequences, information was extracted from UniProtKB/Swiss-Prot , Kyoto Encyclopedia of Genes and Genomes (KEGG), and Gene Ontology (GO). GO and KEGG enrichment analyses were carried out with the Fisher’s exact test, and FDR correction for multiple testing was also performed. Enriched GO and Kegg pathways were nominally statistically significant at the p<0.05 level.

1. Wisniewski, J.R., et al., Universal sample preparation method for proteome analysis. Nat Methods, 2009. 6(5): p. 359-62.
2. Tyanova S, Temu T, Sinitcyn P. The Perseus computational platform for comprehensive analysis of (prote)omics data. 2016;13(9):731-740.
3. Boutet E, Lieberherr D, Tognolli M, et al. UniProtKB/Swiss-Prot, the Manually Annotated Section of the UniProt KnowledgeBase: How to Use the Entry View. Methods in molecular biology (Clifton, NJ) 2016;1374:23-54.
4. Kanehisa M, Goto S, Sato Y, Furumichi M, Tanabe M. KEGG for integration and interpretation of large-scale molecular data sets. Nucleic acids research 2012;40(Database issue):D109-114.
5. Ashburner M, Ball CA, Blake JA, et al. Gene ontology: tool for the unification of biology. The Gene Ontology Consortium. Nature genetics 2000;25(1):25-29.
6. Kohl M, Wiese S, Warscheid B. Cytoscape: software for visualization and analysis of biological networks. Methods in molecular biology (Clifton, NJ) 2011;696:291-303.
